# Supplementary material for: Open access policies of leading medical journals: a cross-sectional study
Source: BMJ Open. 2019 Jun 20;9(6):e028655. doi: 10.1136/bmjopen-2018-028655 (PMC6596940; doi:10.1136/bmjopen-2018-028655)
Supplement: Supplementary file 1 [file bmjopen-2018-028655supp001.pdf]

Supplemental information

Table S1 Definitions of CC licences commonly used by medical journals[17]

Type	Definition
**CC BY**	Free to distribute and adapt the original work, even commercially, if the original creation and authors are credited
**CC BY-NC**	Free to adapt the original work non-commercially and, although derivative works must also acknowledge the authors and be non-commercial, they do not have to be licensed on the same terms
**CC BY-NC-ND**	Free to download the original work and share it if the authors are credited, but the work cannot be adapted or used commercially

CC, Creative Commons; CC BY, Creative Commons Attribution; NC, Non-Commercial; ND, No Derivatives.

Patients' perspectives

After reviewing a draft of our manuscript, Alan Thomas, a patient advocate for rare diseases, shared his experiences. He said, "I regularly encounter paywalls while trying to find high quality and important information on a rare disease for myself and to share with other patients." Because patients can build up substantial understanding during a lifetime living with their rare disease, they are often able to read and understand articles in peer-reviewed journals. However, "when we hit a paywall, we typically give up and look for information elsewhere," he said. Elizabeth Kinder, a patient with multiple sclerosis, highlighted the importance of patients knowing about the latest research to make informed choices about treatments. She said "It is absolutely essential that validated scientific findings are as easily and freely available as all the dangerous false-hope-fake-news. A friend of mine spent his life savings on a pseudo-scientific procedure and after a brief respite, was considerably more disabled than before. And broke. Which increased the negative impact on his health. If publishers refuse to share information they are as immoral as those snake oil salesman and as dangerous to public health. Patients like me have to become our own experts. Faced with a steadily crippling disease like multiple sclerosis, it is not just in our own interests but in the interests of society as a whole that we are able to work, to contribute, to play our part, and for our families to do so, to not become our carers."

Elizabeth Kinder is mindful of the risks she took when taking part in a clinical trial, saying “I think ‘Sudden death’ was no. 3 in the long list of side effects on which I signed off).” She believes that “by withholding free access to research findings, journals are scorning the funders, the researchers, and people like me (patients) who risk their lives participating in a clinical trial”. Alan Thomas noted that many patients do not realize that scientific content is put behind paywalls by the journals in which it is published, and that they could misconstrue such information as being hidden by pharmaceutical companies or others. He wanted to encourage pharmaceutical companies to be more open and collaborative by making their research open access. He said, “open access to medical documents enables communication *with* patients as well as *to* patients, and is vital to patients’ wellbeing and peace of mind”.
