# Supplementary material for: Open access policies of leading medical journals: a cross-sectional study
Source: BMJ Open. 2019 Jun 20;9(6):e028655. doi: 10.1136/bmjopen-2018-028655 (PMC6596940; doi:10.1136/bmjopen-2018-028655)
Supplement: Author's manuscript [file bmjopen-2018-028655.draft_revisions.pdf]

BMJ Open

BMJ Open is committed to open peer review. As part of this commitment we make the peer review history of every article we publish publicly available.

When an article is published we post the peer reviewers' comments and the authors' responses online. We also post the versions of the paper that were used during peer review. These are the versions that the peer review comments apply to.

The versions of the paper that follow are the versions that were submitted during the peer review process. They are not the versions of record or the final published versions. They should not be cited or distributed as the published version of this manuscript.

BMJ Open is an open access journal and the full, final, typeset and author-corrected version of record of the manuscript is available on our site with no access controls, subscription charges or pay-per-view fees (http://bmjopen.bmj.com).

If you have any questions on BMJ Open's open peer review process please email info.bmjopen@bmj.com

Open access policies of leading medical journals: a cross-sectional study

Journal:	*BMJ Open*
Manuscript ID	bmjopen-2018-028655
Article Type:	Research
Date Submitted by the Author:	18-Dec-2018

Complete List of Authors:	Ellison, Tim; PharmaGenesis London<br>Koder, Tim; Oxford PharmaGenesis Ltd<br>Schmidt, Laura; Oxford PharmaGenesis Ltd<br>Williams, Amy; PharmaGenesis London<br>Winchester, Chris; Oxford PharmaGenesis Ltd

Keywords:	article processing charges, CC BY, Creative Commons, funding, open access, pharmaceutical

SCHOLARONE™ Manuscripts

Open access policies of leading medical journals: a cross-sectional study

Authors:
Tim S Ellison[1] (https://orcid.org/0000-0003-0307-725X),
Tim Koder[2] (https://orcid.org/0000-0001-6152-7365),
Laura Schmidt[2] (https://orcid.org/0000-0001-6117-781X),
Amy Williams[1] (https://orcid.org/0000-0002-9354-6402),
Christopher C Winchester[2] (https://orcid.org/0000-0003-3267-3990)

[1]PharmaGenesis London, London, UK
[2]Oxford PharmaGenesis, Oxford, UK

Correspondence to:
Dr Tim Ellison
Address: PharmaGenesis London, 9 Whitehall, 4th Floor, London, SW1A 2DD
Email: tim.ellison@pharmagenesis.com
Phone: +44 203 675 7065

Keywords: article processing charges, CC BY, Creative Commons, funding, open access, pharmaceutical, industry, commercial, disclosure
Word count (excluding abstract, references and figures and tables): 2827
Abstract word count: 299
Figures and tables: 6 (excluding 2 supplemental tables)
References: 45

For peer review only

ABSTRACT

Objectives

Academic and not-for-profit research funders are increasingly requiring that the research they fund must be published open access, with some insisting on publishing with a Creative Commons Attribution (CC BY) licence to allow the broadest possible use. We set out to clarify the open access variants provided by leading medical journals for research in general and industry-funded research in particular, and record the availability of the CC BY licence for commercially-funded research.

Methods

We identified medical journals with a 2015 impact factor of ≥15.0 on 24 May 2017, then excluded from the analysis journals that only publish review articles. Between 29 June 2017 and 26 July 2017, we collected information about each journal's open access policies from their websites and/or by email contact. We contacted the journals by email again between 6 December 2017 and 2 January 2018 to confirm our findings.

Results

Thirty-five medical journals publishing original research from 13 publishers were included in the analysis. All 35 journals offered some form of open access with varying embargo periods of up to 12 months. Of these journals, 21 (60%) provided immediate open access with a CC BY licence under certain circumstances (e.g. to specific research funders). Of these 21, 20 only offered a CC BY licence to authors funded by non-commercial organizations and one offered this option to funders who required it.

Conclusions

Most leading medical journals do not offer to authors reporting commercially funded research an open access licence that allows unrestricted sharing and adaptation of the published material. The journals' policies are therefore not aligned with open access declarations and guidelines. Commercial research funders lag behind academic funders in the development of mandatory open access policies, and it is

For peer review only - http://bmjopen.bmj.com/site/about/guidelines.xhtml

time for them to work with publishers to advance the dissemination of the research they fund.

For peer review only

For peer review only - http://bmjopen.bmj.com/site/about/guidelines.xhtml

Strengths and limitations of this study

- This manuscript includes a systematic analysis of open access policies of journals with a high impact factor, including society-owned journals, from multiple publishers.
- The open access policies of all journals analysed were clarified, and confirmation of our findings was received by email from 97% of the contacted journals.
- Open access policies of the journals and publishers analysed are subject to change, so the information presented here may not be current.
- By selecting journals with a high impact factor, our analysis does not include prestigious journals from specialized therapy areas and regional or non-English language journals, which may have lower impact factors.
- Although our study covers only a small number of journals, extending such a manual analysis to a greater number of journals without loss of detail and verification of all results would be cumbersome and inefficient by relying on traditional analysis tools.

For peer review only - http://bmjopen.bmj.com/site/about/guidelines.xhtml

For peer review only

INTRODUCTION

Hundreds of billions of US dollars are invested in medical research by governments, charities and commercial organizations each year, with the aim of extending and improving human lives.[1] Publication plays an important role in the dissemination of scientific innovation.[2 3] However, translation of medical research into clinical practice is slow; one study has suggested that it takes an average of 17 years for research evidence to reach 50% adoption in clinical practice, with the longest delays occurring after successful publication of clinical trial results.[2 3]

Open access publishing has the potential to improve innovation and speed up its adoption. Complete access to research literature encourages viewing of more articles than partial access,[4 5] and open access articles appear to be downloaded more often and receive more citations than subscription articles, indicating a greater academic impact.[6-9] There is also evidence suggesting that open access articles have a broader societal impact based on Altmetric data that measure the amount of attention publications receive in the news media and social communication channels.[9-11] Depending on the restrictiveness of its licensing, open access can facilitate public and commercial reuse of research results, which is beneficial for collaboration, education and innovation.[9] Furthermore, enabling access to the full text of research articles increases transparency, which benefits the public by helping both doctors and patients to find complete and current evidence to inform treatment decisions, and by preventing potentially harmful decisions being made based on the abstracts of paywalled articles.[9 12-14] It is worth noting that the publishing model used by a journal (i.e. open access or subscription) has no impact on the quality of articles published.[15 16]

‘Open access’ is a broad term that encompasses a range of definitions, from ‘free-to-read’ (full text available to read on demand, without charge to the reader) to ‘free-to-read and reuse’ (with the additional ability to reuse text, tables and figures in different formats). When a journal offers open access, it has wide leeway in the choice of policy or policies it will apply, using one of the Creative Commons licences that allow reuse under specific terms, or offering free-to-read access without a licence.[17]

For peer review only - http://bmjopen.bmj.com/site/about/guidelines.xhtml

The Open Access Scholarly Publishers Association and the Budapest Open Access Initiative recommend the Creative Commons Attribution (CC BY) licence because it allows sharing and adaptation of published materials for any purposes (both commercial and non-commercial), subject only to attribution of the original source.[17-19] Common alternatives to the CC BY licence include CC BY Non-Commercial (CC BY-NC), which restricts commercial reuse; CC BY No Derivatives (CC BY-ND), which restricts adaptation; and CC BY-NC-ND, which restricts both (table S1).[17 20]

Open access with a Creative Commons licence is typically facilitated by article processing charges. Following payment of such a charge by the research author, institution or funder, open access articles with a Creative Commons licence are usually made available on the journal's website at the time of publication in the publisher's typeset format (Version of Record). Open access articles that do not include a Creative Commons licence at the time of publication typically involve an embargo period before the published articles are freely accessible and may allow access only to the accepted manuscript (a version that has not been edited and typeset by the journal), which is made available on the author's institutional website or on PubMed Central/Europe PubMed Central without a requirement for payment.

There has been an increasing trend towards open access publishing over the last 20 years, and almost 50% of articles were published open access in 2015.[8] Many academic and not-for-profit research funders now require the research they fund to be published open access.[9 21-26] Prominently, the Wellcome Trust and the Bill & Melinda Gates Foundation insist on publishing with a CC BY licence to allow the broadest possible use.[21 23] Commercial research funders, which fund approximately half of all medical research,[1 27 28] have been more hesitant to require open access publishing but now commonly pay for open access when the option is available.[24] In January 2018, Shire became the first commercial research funder to require all research manuscripts it funds to be published open access.[29 30] At present, no commercial funder requires open access publishing with a CC BY licence.

We set out to clarify the open access variants provided by leading medical journals for research, in general and industry-funded research in particular, and establish the availability of the CC BY licence for industry-funded research.

For peer review only - http://bmjopen.bmj.com/site/about/guidelines.xhtml

METHODS

Using Journal Selector (Sylogent, Newtown, PA, USA), we identified medical journals with a 2015 impact factor of at least 15.0 (accurate on 24 May 2017). To focus on journals publishing original medical research, we excluded journals that only publish review articles. We collected information on the open access variants provided by the included journals from their websites and by email contact when information was missing or unclear, making up to three attempts between 29 June 2017 and 26 July 2017.

For each journal, we recorded the following information:

- for immediate open access, whether a CC BY licence or other Creative Commons licence was provided
- for delayed open access, the length of embargo period for open access
- for both immediate and delayed open access, which version of the article would be available (published Version of Record or accepted).

For journals that provided a CC BY licence, we additionally collected information on:

- the requirements for obtaining a CC BY licence (e.g. dependence on funding source)
- article processing charges.

Between 6 December 2017 and 2 January 2018, we emailed the journals' editorial offices requesting confirmation of our findings (table S2). Once open access variants were recorded, we categorized the most open variant provided by each included journal using our own classification, as shown in table 1.

For peer review only

RESULTS

Included journals

Fifty-three journals listed in the Journal Selector database had a 2015 impact factor of at least 15.0 (figure 1). After 16 review journals and two non-medical journals were excluded, 35 journals from 13 publishers were included in this analysis. Of the 15 journals that were contacted to clarify information that was missing or unclear, 14 replied with clarification. Once all information was collected and tabulated, we received confirmation of our findings from 34 (97%) of the 35 journals.

Open access variants offered

Proportions of journals in each category of the most open variant of open access are shown in figure 2A. Immediate open access with a Creative Commons licence was provided by 21 (60%) of the 35 journals analysed. The types of Creative Commons licence available from these 21 journals under different circumstances were: CC BY from 21 journals (100%); CC BY-NC from 4 journals (19% of all journals offering CC BY); and CC BY-NC-ND from 18 journals (86% of all journals offering CC BY).

When the 35 analysed journals were categorized by impact factor, immediate open access with a CC BY or other Creative Commons licence was provided by 10 (66%) of the 15 journals with an impact factor between 15.0 and 19.9, and 3 (30%) of the 10 journals with an impact factor over 30.0 (figure 2B).

All 14 journals, from six publishers, that did not provide open access with a Creative Commons licence provided access to different versions of the article either immediately, after a 6-month embargo period or after a 12-month embargo period under different circumstances (table 2).

For peer review only

The cost of open access with a CC BY licence

Of the 21 journals that offered a CC BY licence, 19 (90%) disclosed article processing charges on their websites. Across these journals, charges ranged from USD $3000 to $5000; the most common article processing charge was $5000 (in 13 [62%] of journals; figure 3). Details of the fees charged by the remaining two journals (10%) were not available from their websites because the details were only provided when the article was accepted.

Relationship between funding source and the availability of open access variants

Table 3 shows the open access policies of the journals offering open access with a CC BY licence. Of the 21 journals listed, 20 journals allowed open access with a CC BY licence for research funded by specific non-commercial organizations, and only *The BMJ* offered it to organizations who required it, regardless of the nature of the funding source.

DISCUSSION

Here, we present a systematic analysis of open access policies of journals with a high impact factor, including society-owned journals, from multiple publishers. We met our objective to clarify the open access policies of all journals analysed and received confirmation of our findings by email from 97% of the contacted journals. We found that all leading medical journals in this study provided some form of open access, but there was little consistency across their policies. Over half of the included journals provided a CC BY licence; however, with the exception of one journal, this option was only available only to authors funded by non-commercial organizations. One journal (*The BMJ*) allowed authors to obtain a CC BY licence when the work was supported by funders who required its use. Therefore, if pharmaceutical companies had a policy that required open access with a CC BY licence, the *The BMJ* would be suitable, and other journals might be inclined to change their policy.

Limitations of this study are that we investigated journals listed in the Journal Selector database with an impact factor of at least 15.0, and that, because impact

For peer review only - http://bmjopen.bmj.com/site/about/guidelines.xhtml

factors and the open access policies of journals and publishers are subject to change, the information may not be current. Furthermore, by selecting journals with a high impact factor, our analysis does not include prestigious journals from specialized therapy areas and regional or non-English language journals, which may have lower impact factors. Although our study covers only a small number of journals, extending such a manual analysis to a greater number of journals without loss of detail and verification of all results would be cumbersome and inefficient by relying on traditional analysis tools. If more extensive mining of journal (meta)data becomes feasible, however, this study could be repeated for a bigger cohort of journals.

To our knowledge, this is the first report showing that the availability of open access options depends on the source of funding. Limitations on the availability of the CC BY licence depending on the research funder are not in line with statements such as the Budapest Declaration,[18] the Berlin Declaration[31] and the Bethesda Statement,[32] which aim to provide end users with immediate access to research articles and to give them the opportunity to reuse material without restrictions. Furthermore, placing restrictions on access to medical research owing to its source of funding is not in line with the key principles of human research ethics laid out in the Declaration of Helsinki.[33]

Good Publication Practice 3 (GPP3) guidelines state that authors should take responsibility for the way research findings are published.[34] In line with these recommendations, pharmaceutical companies can and, we believe, should advise authors to reach a consensus on which journal to publish with, to avoid predatory journals, and to adhere to sponsor guidelines and regulations. In the authors' experience, some pharmaceutical companies already have internal guidelines recommending open access publishing, and one (Shire) now requires it.

Our research shows that one-third of the journals with a high impact factor do not offer immediate access to the published version of a manuscript upon publication, even though the open access policies of many funders with respect to embargo periods echo the recommendations set out by open access declarations worldwide.[18, 21-23, 26, 31, 32, 35] Of note, Horizon 2020, which is supported by the European Research

For peer review only - http://bmjopen.bmj.com/site/about/guidelines.xhtml

Council, requires its beneficiaries to make publications open access no later than 6 months after the official publication date and to make every effort to allow for maximum reuse of the materials, whether that be copying, distributing, searching, linking, crawling, mining or some other use.[36 37] Furthermore, cOAlition S, a group of national research funders with the support of the European Commission and the European Council, has committed to Plan S, the key principle of which is that scientific publications on research funded by participating national and European funders must be published open access by 2020.[35] Under the terms of Plan S, authors must retain copyright of their publication with no restrictions, and all publications must be published under an immediate open licence (preferably CC BY) that fulfils the requirements defined by the Berlin Declaration.[35 36]

Policies vary between publishers but also across journals at the same publisher, and this is also the case for journals not included in this analysis, as shown, for example, by Taylor & Francis in their table of the policies of all their journals.[38] Differences in policy have many underlying factors, including the choices of the journals' academic editorial boards and societies. A potential disincentive to publishers offering CC BY licences to the pharmaceutical industry is the revenue generated from copyright fees and reprints. Permission to reproduce copyrighted materials can cost hundreds or even thousands of dollars; for example, the permission fee requested for reuse of a single table containing 40 words in the journal *American Family Physician* was $4400.[39] Reprints can cost significantly more than permissions charges; for example, reprint sales from a single clinical trial can total $1 million or more, with a large profit margin.[40]

Research by Lundh *et al.*[41] aimed to quantify reprint revenues as a proportion of journal income. Of the six journals investigated, the two European journals, *The BMJ* and *The Lancet*, disclosed the information requested. The editors of the US journals *Archives of Internal Medicine*, *Annals of Internal Medicine*, *JAMA* and the *New England Journal of Medicine* did not provide the data. For *The BMJ*, reprint revenues constituted 3% of its overall income; *The Lancet* obtained 41% of its revenue from reprints.[41] In *The Lancet*, industry-funded publications constituted a large proportion of highly reprinted articles (63/88) compared with a sample of control articles from the same journal (23/88).[42] The generation of revenue for publishers from the selling

For peer review only - http://bmjopen.bmj.com/site/about/guidelines.xhtml

of reprints leaves publishers open to the criticism that bias can be introduced into editorial decisions.[41] This concern could be addressed by a transition to open access publishing exclusively with a CC BY licence. However, such a transition may need to be managed.

Two of the journals included in our analysis, *Science* and *Science Translational Medicine*, both published by the American Association for the Advancement of Science, do not disclose article processing charges on their websites;[43] instead, they provide this information upon their acceptance of an article. This practice does not comply with the Directory of Open Access Journals guidelines,[44] which state that processing fees must be stated clearly on journal websites in a place that is easy to find for potential authors prior to submitting their manuscript. The practice is also common among predatory journals, potentially reinforcing perceptions held by some academics of the association between open access and predatory publishing.

We found that the open access policies of some journals precluded commercially funded research from being published open access, even after an embargo period and without a Creative Commons licence. Further analyses could therefore be undertaken to clarify the proportion of journals with this policy and the rationale behind this position. Future research could also focus on a larger cohort of journals than the current study, or on journals from a specific therapy area, to further clarify the use of open access variants in the medical publications landscape.

CONCLUSIONS

The CC BY licence is recommended by open access declarations and funders of research as the optimal open access licence. Our analysis shows that although journals with a high impact factor provide some form of open access, they restrict commercially funded research from being published with the CC BY licence. Approximately half of all medical research is funded by the pharmaceutical industry,[1 27 28] meaning that the research output cannot be reused or built upon if it is published in journals with a high impact factor without payment of additional fees, hampering research innovation and collaboration. However, there are concerns that

For peer review only - http://bmjopen.bmj.com/site/about/guidelines.xhtml

a rapid transition to publishing exclusively with a CC BY licence will be difficult, given current processes and business models in scientific publishing.

The idea that open access to research articles is beneficial to all stakeholders in medical research and publishing is compelling. Open access publishing facilitates faster and more thorough disclosure of research, removes barriers for groups conducting systematic reviews, increases both the citation counts and Altmetric scores of publications, and benefits patient health by improving informed decision-making by doctors and patients.[9] Commercial research funders lag behind non-commercial funders in the implementation of open access policies, and it is time for them to close the gap. Pharmaceutical companies should make clear their open access requirements, for example in a unified position statement, ideally aligned with open access declarations,[18 31 32] the Horizon 2020 programme and Plan S,[35-37] and the International Committee of Medical Journal Editors[45] and GPP3[34] guidelines, and then work together with publishers to realise the ultimate goal of improved access to medical research for all.

For peer review only

Acknowledgements

Robert Kiley (https://orcid.org/0000-0003-4733-2558) is Head of Open Research at the Wellcome Trust, London, UK, and contributed to the review of this manuscript. Paul Farrow (https://orcid.org/0000-0002-0569-9688) is an employee of Oxford PharmaGenesis, Oxford, UK, and contributed significantly to the review of this manuscript. Sarah Stokes (https://orcid.org/0000-0002-8761-8588) and Velissaria Vanna are employees of Oxford PharmaGenesis, Oxford, UK, and contributed to the review and editing of this manuscript. This work was presented as a poster at both the European Meeting of the International Society for Medical Publication Professionals (ISMPP) on 23 January 2018 and the Annual Meeting of ISMPP on 2 May 2018, and was posted to bioRxiv as a preprint on 22 January 2018 (https://www.biorxiv.org/content/early/2018/01/22/250613).

Funding statement

This research was funded by Oxford PharmaGenesis.

Competing interests

Tim Ellison, Tim Koder, Amy Williams and Chris Winchester are employees of Oxford PharmaGenesis, Oxford, UK. At the time of the research and writing of this manuscript, Laura Schmidt was an employee of Oxford PharmaGenesis, Oxford, UK. Chris Winchester is also a Director and a shareholder of Oxford PharmaGenesis Holdings Ltd.

Author contributions

Conceptualization, project administration, TE (https://orcid.org/0000-0003-0307-725X), TK (https://orcid.org/0000-0001-6152-7365), LS (https://orcid.org/0000-0001-6117-781X), AW (https://orcid.org/0000-0002-9354-6402); methodology, resources, investigation, formal analysis, TE; writing – original draft, TE and LS; visualization, TE; writing – review and editing, TE, TK, LS, AW, CW (https://orcid.org/0000-0003-3267-3990); supervision, TK, LS.

For peer review only - http://bmjopen.bmj.com/site/about/guidelines.xhtml

References

1. Moses H, 3rd, Matheson DH, Cairns-Smith S*, et al.* The anatomy of medical research: US and international comparisons. *JAMA* 2015;313(2):174–89. doi: 10.1001/jama.2014.15939.

2. Balas EA, Boren SA. Managing clinical knowledge for health care improvement. *Yearb Med Inform* 2000(1):65–70.

3. Balas EA, Chapman WW. Road map for diffusion of innovation in health care. *Health Aff (Millwood)* 2018;37(2):198–204. doi: 10.1377/hlthaff.2017.1155.

4. Maggio LA, Moorhead LL, Willinsky JM. Qualitative study of physicians' varied uses of biomedical research in the USA. *BMJ Open* 2016;6(11):e012846. doi: 10.1136/bmjopen-2016-012846.

5. Moorhead LL, Holzmeyer C, Maggio LA*, et al.* In an age of open access to research policies: physician and public health NGO staff research use and policy awareness. *PLoS One* 2015;10(7):e0129708. doi: 10.1371/journal.pone.0129708.

6. Davis PM, Lewenstein BV, Simon DH*, et al.* Open access publishing, article downloads, and citations: randomised controlled trial. *BMJ* 2008;337:a568. doi: 10.1136/bmj.a568.

7. Ottaviani J. The post-embargo open access citation advantage: it exists (probably), its modest (usually), and the rich get richer (of course). *PLoS One* 2016;11(8):e0159614. doi: 10.1371/journal.pone.0159614.

8. Piwowar H, Priem J, Lariviere V*, et al.* The state of OA: a large-scale analysis of the prevalence and impact of Open Access articles. *PeerJ* 2018;6:e4375. doi: 10.7717/peerj.4375.

9. Tennant JP, Waldner F, Jacques DC*, et al.* The academic, economic and societal impacts of open access: an evidence-based review. *F1000Res* 2016;5:632. doi: 10.12688/f1000research.8460.3.

10. Wang X, Liu C, Mao W*, et al.* The open access advantage considering citation, article usage and social media attention. *Scientometrics* 2015;103(2):555–64. doi: 10.1007/s11192-015-1547-0.

For peer review only

For peer review only - http://bmjopen.bmj.com/site/about/guidelines.xhtml

11. Allen HG, Stanton TR, Di Pietro F*, et al.* Social media release increases dissemination of original articles in the clinical pain sciences. *PLoS One* 2013;8(7):e68914. doi: 10.1371/journal.pone.0068914.

12. Hopewell S, Clarke M, Moher D*, et al.* CONSORT for reporting randomised trials in journal and conference abstracts. *Lancet* 2008;371(9609):281–3. doi: 10.1016/S0140-6736(07)61835-2.

13. Barbour V, Chinnock P, Cohen B*, et al.* The impact of open access upon public health. *Bull World Health Organ* 2006;84(5):337–424.

14. Leung PTM, Macdonald EM, Stanbrook MB*, et al.* A 1980 letter on the risk of opioid addiction. *N Engl J Med* 2017;376(22):2194–95. doi: 10.1056/NEJMc1700150.

15. Pastorino R, Milovanovic S, Stojanovic J*, et al.* Quality assessment of studies published in open access and subscription journals: results of a systematic evaluation. *PLoS One* 2016;11(5):e0154217. doi: 10.1371/journal.pone.0154217.

16. Tahim A, Bansal H, Goodson AM*, et al.* Open access publishing: a study of current practice in oral and maxillofacial surgery research. *J Maxillofac Oral Surg* 2016;15(4):517-20. doi: 10.1007/s12663-016-0898-2.

17. Creative Commons. About The Licenses: Creative Commons; 2017. Available from: https://creativecommons.org/licenses/ (Accessed 29 October 2018).

18. Budapest Open Access Initiative. Ten years on from the Budapest Open Access Initiative: setting the default to open 2012. Available from: http://www.budapestopenaccessinitiative.org/boai-10-recommendations (Accessed 2 January 2018).

19. Williams L. Best practices in licensing and attribution: What you need to know: OASPA; 2016. Available from: https://oaspa.org/best-practices-licensing-attribution-need-to-know/ (Accessed 3 January 2018).

20. Stodden V, Leisch F, Peng RD. Implementing Reproducible Research: Taylor & Francis 2014.

21. Open access policy: Wellcome Trust. Available from: https://wellcome.ac.uk/funding/managing-grant/open-access-policy (Accessed 3 January 2018).

22. Charity Open Access Fund. COAF guidelines: Charity Open Access Fund; 2017. Available from: https://wellcome.ac.uk/funding/managing-grant/charity-open-access-fund (Accessed 2 January 2018).

For peer review only

For peer review only - http://bmjopen.bmj.com/site/about/guidelines.xhtml

23. Bill & Melinda Gates Foundation. How We Work: Bill & Melinda Gates Foundation Open Access Policy 2015. Available from: https://www.gatesfoundation.org/How-We-Work/General-Information/Open-Access-Policy (Accessed 3 January 2018).

24. Collins E. Publishing priorities of biomedical research funders. *BMJ Open* 2013;3(10):e004171. doi: 10.1136/bmjopen-2013-004171.

25. Marchington J, Panayi A, Baronikova S*, et al.* Open access licences: what drives publisher options? Suppl 1 (26)2017. Available from: http://www.caudex.com/downloads/OA_survey_EU_ISMPP_2017_poster_15.pdf (Accessed 3 January 2018).

26. Medical Research Council UK. RCUK Policy on Open Access and Supporting Guidance 2017. Available from: http://www.rcuk.ac.uk/documents/documents/rcukopenaccesspolicy-pdf/ (Accessed 2 January 2018).

27. Dorsey ER, de Roulet J, Thompson JP*, et al.* Funding of US biomedical research, 2003–2008. *JAMA* 2010;303(2):137–43. doi: 10.1001/jama.2009.1987.

28. Hakoum MB, Jouni N, Abou-Jaoude EA*, et al.* Characteristics of funding of clinical trials: cross-sectional survey and proposed guidance. *BMJ Open* 2017;7(10):e015997. doi: 10.1136/bmjopen-2017-015997.

29. The MAP Newsletter. Shire announces new open access policy: ISMPP; 2018 updated 20 January 2018. Available from: http://ismpp-newsletter.com/2018/01/30/shire-announces-new-open-access-policy/ (Accessed 20 July 2018).

30. Shire. Shire continues to uphold high standards of ethics and transparency with adoption of open access policy for publication of Shire-supported research 2018. Available from: https://www.shire.com/en/newsroom/2018/january/xajhds (Accessed 16 July 2018).

31. Open access Max-Planck-Gesellschaft. Berlin Declaration on Open Access to Knowledge in the Sciences and Humanities 2003. Available from: https://openaccess.mpg.de/Berlin-Declaration (Accessed 2 January 2018).

32. Brown PO, Cabell D, Chakravarti A*, et al.* Bethesda Statement on Open Access Publishing Chevy Chase, Maryland, US 2003. Available from: http://legacy.earlham.edu/~peters/fos/bethesda.htm (Accessed 3 January 2018).

33. WMA Declaration of Helsinki – Ethical principles for medical research involving human subjects: World Medical Association. Available from:

For peer review only - http://bmjopen.bmj.com/site/about/guidelines.xhtml

https://www.wma.net/policies-post/wma-declaration-of-helsinki-ethical-principles-for-medical-research-involving-human-subjects/ (Accessed 19 January) 2018.

34. Battisti WP, Wager E, Baltzer L*, et al.* Good Publication Practice for Communicating Company-Sponsored Medical Research: GPP3. *Ann Intern Med* 2015;163(6):461–4. doi: 10.7326/M15-0288.

35. Science Europe. cOAlition S 2018. Available from: https://www.scienceeurope.org/coalition-s/ (Accessed 29 October 2018).

36. Guidelines to the rules on open access to scientific publications and open access to research data in Horizon 2020 (Version 3.2): EUROPEAN COMMISSION Directorate-General for Research & Innovation; 2017. 11. Available from: http://ec.europa.eu/research/participants/data/ref/h2020/grants_manual/hi/oa_pilot/h2020-hi-oa-pilot-guide_en.pdf (Accessed 17 January 2018).

37. Guidelines on the Implementation of Open Access to Scientific Publications and Research Data in projects supported by the European Research Council under Horizon 2020: European Research Council 2017. Available from: https://erc.europa.eu/sites/default/files/document/file/ERC%20Open%20Access%20guidelines-Version%201.1._10.04.2017.pdf (Accessed 3 January 2018).

38. Author Services Supporting Taylor & Francis authors. Open access options: Taylor & Francis. Available from: https://authorservices.taylorandfrancis.com/journal-list/ (Accessed 20 July 2018).

39. Siwek J. Permission fees for reproducing tables in journal articles are exorbitant. *BMJ* 2015;351:h5128. doi: 10.1136/bmj.h5128.

40. Smith R. Medical journals and pharmaceutical companies: uneasy bedfellows. *BMJ* 2003;326(7400):1202–5. doi: 10.1136/bmj.326.7400.1202.

41. Lundh A, Barbateskovic M, Hrobjartsson A*, et al.* Conflicts of interest at medical journals: the influence of industry-supported randomised trials on journal impact factors and revenue – cohort study. *PLoS Med* 2010;7(10):e1000354. doi: 10.1371/journal.pmed.1000354.

42. Handel AE, Patel SV, Pakpoor J*, et al.* High reprint orders in medical journals and pharmaceutical industry funding: case-control study. *BMJ* 2012;344:e4212. doi: 10.1136/bmj.e4212.

43. Science Journals: editorial policies: *Science*. Available from: http://www.sciencemag.org/authors/science-journals-editorial-policies (Accessed 14 September) 2018.

For peer review only - http://bmjopen.bmj.com/site/about/guidelines.xhtml

44. Principles of transparency and best practice in scholarly publishing: DOAJ. Available from: https://blog.doaj.org/2018/01/15/principles-of-transparency-and-best-practice-in-scholarly-publishing-version-3/ (Accessed 18 January) 2018.

45. International Committee of Medical Journal Editors. Recommendations for the conduct, reporting, editing, and publication of scholarly work in medical journals: International Committee of Medical Journal Editors; 2017. Available from: http://www.icmje.org/icmje-recommendations.pdf (Accessed 2 January 2017).

For peer review only

Tables

Table 1 Categorization of journals based on the most open variant of open access offered.

Category	Version of article available	Embargo period	CC BY licence offered by the journal?
**1**	Published	None	Yes
**2**	Published	None	No
**3**	Published/accepted	≤ 12 months	No

CC BY, Creative Commons Attribution.

For peer review only - http://bmjopen.bmj.com/site/about/guidelines.xhtml

Table 2 Access policies of journals with high impact factors that do not provide open access with Creative Commons licences.

Publisher	Journals included (n=14)	Open access variants available*	
		**Embargo period**	**Version of article available**
**American Association for Cancer Research journals**	*Cancer Discov*	None	VoR†
		6–12 months	Accepted
**American College of Physicians**	*Ann Intern Med*	6 months	Accepted
**American Medical Association**	*JAMA*	None	VoR‡
		6 months	VoR
**Massachusetts Medical Society**	*N Engl J Med*	6 months	VoR
**Nature Publishing Group**	*Nature*; *Nat Biotechnol*; *Nat Cell Biol*; *Nat Genet*; *Nat Immunol*; *Nat Mater*; *Nat Med*; *Nat Methods*; *Nat Neurosci*	6 months	Accepted
**Wiley-Blackwell**	*World Psychiatry*	12 months	Accepted

*Available under the terms specified on the journal website.

†Upon payment of USD $3500 AuthorChoice fee.

‡Available to read on JAMA Network Reader.

VoR, version of record.

For peer review only - http://bmjopen.bmj.com/site/about/guidelines.xhtml

Table 3. Open access policies of journals with high impact factors that offer immediate open access with the CC BY licence (n=21) Creative Commons licences are shaded.

**Publisher**	**Journals included (n=21)**	**Open access variants available***			**Funding requirements for obtaining open access with a CC BY licence**
		**Embargo period**	**Creative Commons licence**	**Version of article available**	
**American Association for the Advancement of Science**	*Science*; *Sci Transl Med*	None	CC BY	VoR	The American Association for the Advancement of Science "will allow authors funded by the Bill & Melinda Gates Foundation to publish their research with a CC BY licence"†
		None	None	Accepted	
		6 months	None	Accepted	
		12 months	None	VoR	
**American Society of Clinical Oncology**	*J Clin Oncol*	None	CC BY<br>CC BY-NC-ND	VoR	Creative Commons licences available only if funders are "academic institutions, not-for-profit organizations, philanthropic foundations or government agencies"
		6 months	None	VoR	
		12 months	None	VoR	
**BMJ Publishing Group**	*BMJ*	None	CC BY<br>CC BY-NC	VoR	CC BY licence available for authors "where the funder requires it"
**Cell Press**	*Cancer Cell*; *Cell*;	None	CC BY	VoR	Creative Commons licences "available only to

For peer review only - http://bmjopen.bmj.com/site/about/guidelines.xhtml

	Cell Metab; *Cell Stem Cell*; *Immunity*		CC BY-NC-ND		authors covered by a funding body agreement” (these non-commercial funding bodies are listed on the journal websites)
		12 months	None	Accepted	
**Elsevier**	*Eur Urol*; *Gastroenterology*; *J Am Coll Cardiol; Lancet*; *Lancet Diabetes Endocrinol*; *Lancet Infect Dis*; *Lancet Oncol*; *Lancet Neurol*; *Lancet Respir Med*	None	CC BY<br>CC BY-NC-ND	VoR	Creative Commons licences are available to authors funded by specific funding bodies (these non-commercial funding bodies are listed on the journal websites)<br><br>Elsevier has established agreements and developed policies to allow authors who publish in Elsevier journals to comply with manuscript archiving requirements of various funding bodies (these non-commercial funding bodies are listed on the journal websites)
		6 months	None	VoR	
**European Society of Cardiology**	*Eur Heart J*	None	CC BY<br>CC BY-NC<br>CC BY-NC-ND	VoR	“RCUK/Wellcome Trust-funded authors…can use the CC BY licence for their articles”
		None	None	Accepted	
		12 months	None	Accepted	
**Lippincott Williams &**	*Circulation*	None	CC BY<br>CC BY-NC	VoR	“Note that authors funded by RCUK or the Wellcome Trust may choose the CC BY

For peer review only

**Wilkins**			CC BY-NC-ND		licence if they agree to pay the article processing charge and commercial reuse of the article is not a factor”
		6–12 months	None	Accepted	
**Wiley-Blackwell**	*CA Cancer J Clin*	None	CC BY<br>CC BY-NC<br>CC BY-NC-ND	VoR	“All RCUK and Wellcome Trust-funded authors will be directed to the CC BY licence”
		12–24 months	None	Accepted	

*Available under the terms specified on the journal website.

†The American Association for the Advancement of Science's pilot open access partnership with the Gates Foundation concluded on 30 June 2018.[43]

CC BY, Creative Commons Attribution; NC, Non-Commercial; ND, No Derivatives; RCUK, Research Councils UK; VoR, version of record.

For peer review only

For peer review only - http://bmjopen.bmj.com/site/about/guidelines.xhtml

Figure legends

Figure 1 Flow chart of journals included in this study.

Figure 2 Medical journals categorized by impact factor and their most open variant of open access available (n=35). **(A)** Impact factor, ≥15.0; **(B)** Impact factors, 15.0–19.9, 20.0–29.9 and ≥30.0.

CC BY, Creative Commons Attribution licence.

Figure 3 Article processing charges of journals that offer immediate open access with a CC BY licence (n=21).

*Details on processing fees are provided at acceptance.[43]

For peer review only

For peer review only - http://bmjopen.bmj.com/site/about/guidelines.xhtml

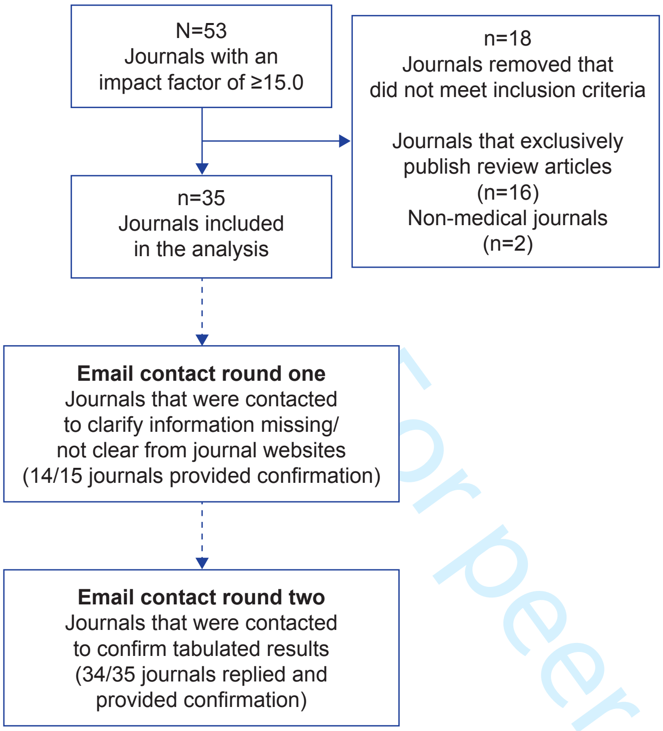

For peer review only

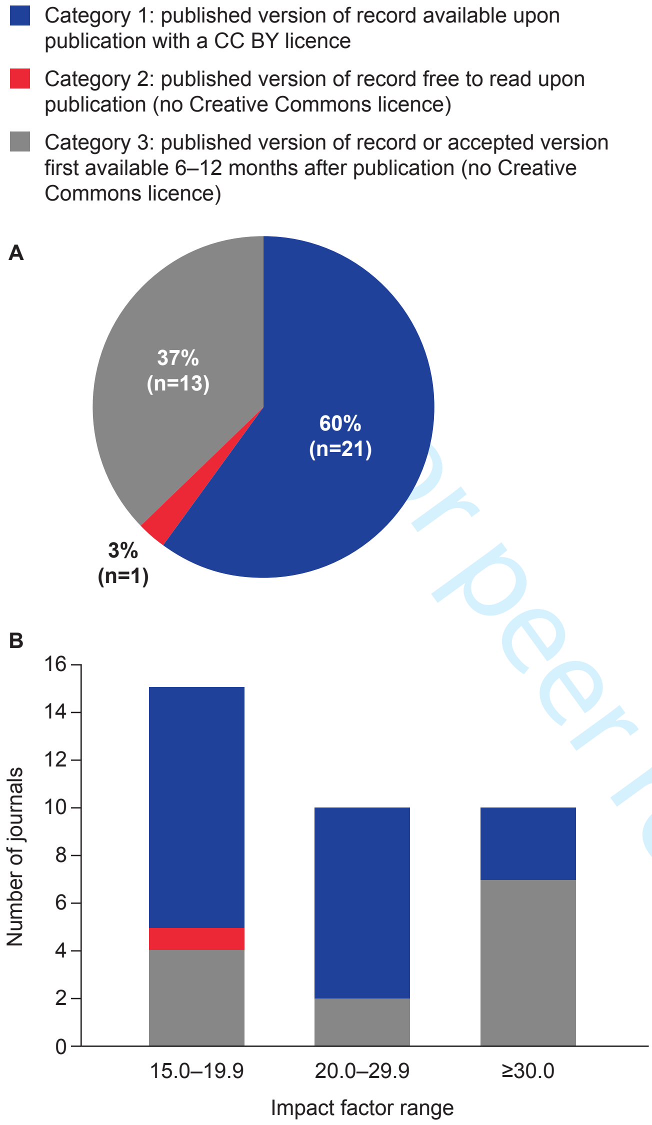

For peer review only

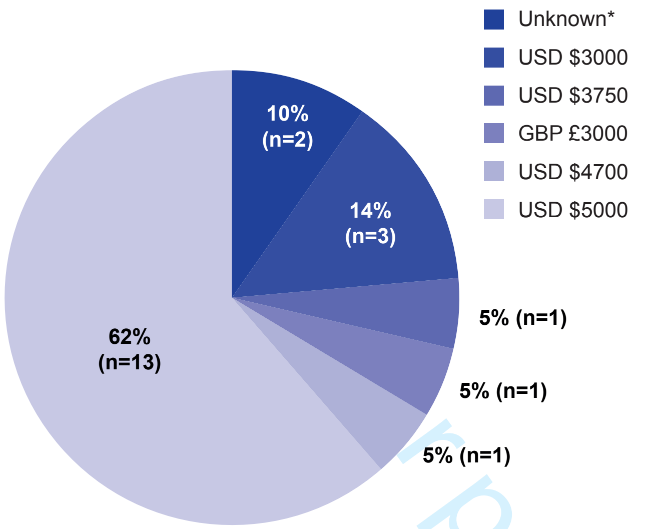

For peer review only

Supplemental information

Table S1 Definitions of CC licences commonly used by medical journals[17]

Type	Definition
**CC BY**	Free to distribute and adapt the original work, even commercially, if the original creation and authors are credited
**CC BY-NC**	Free to adapt the original work non-commercially and, although derivative works must also acknowledge the authors and be non-commercial, they do not have to be licensed on the same terms
**CC BY-NC-ND**	Free to download the original work and share it if the authors are credited, but the work cannot be adapted or used commercially

CC, Creative Commons; CC BY, Creative Commons Attribution; NC, Non-Commercial; ND, No Derivatives.

For peer review only

BMJ Open

Open access policies of leading medical journals: a cross-sectional study

Journal:	*BMJ Open*
Manuscript ID	bmjopen-2018-028655.R1
Article Type:	Research
Date Submitted by the Author:	14-Mar-2019
Complete List of Authors:	Ellison, Tim; PharmaGenesis London<br>Koder, Tim; Oxford PharmaGenesis Ltd<br>Schmidt, Laura; Oxford PharmaGenesis Ltd<br>Williams, Amy; PharmaGenesis London<br>Winchester, Chris; Oxford PharmaGenesis Ltd
**Primary Subject Heading**:	Medical publishing and peer review
Secondary Subject Heading:	Communication
Keywords:	article processing charges, CC BY, Creative Commons, funding, open access, pharmaceutical

SCHOLARONE™
Manuscripts

Open access policies of leading medical journals: a cross-sectional study

Authors:

Tim S Ellison[1] (https://orcid.org/0000-0003-0307-725X),

Tim Koder[2] (https://orcid.org/0000-0001-6152-7365),

Laura Schmidt[2] (https://orcid.org/0000-0001-6117-781X),

Amy Williams[1] (https://orcid.org/0000-0002-9354-6402),

Christopher C Winchester[2] (https://orcid.org/0000-0003-3267-3990)

[1]PharmaGenesis London, London, UK

[2]Oxford PharmaGenesis, Oxford, UK

Correspondence to:

Dr Tim Ellison

Address: PharmaGenesis London, 9 Whitehall, 4th Floor, London, SW1A 2DD

Email: tim.ellison@pharmagenesis.com

Phone: +44 203 675 7065

Keywords: article processing charges, commercial, CC BY, Creative Commons, funding, open access, pharmaceutical.

Word count (excluding abstract, references and figures and tables): 3776

Abstract word count: 300

Figures and tables: 6 (excluding 1 supplemental table)

References: 53

For peer review only

ABSTRACT

Objectives

Academic and not-for-profit research funders are increasingly requiring that the research they fund must be published open access, with some insisting on publishing with a Creative Commons Attribution (CC BY) licence to allow the broadest possible use. We aimed to clarify the open access variants provided by leading medical journals and record the availability of the CC BY licence for commercially funded research.

Methods

We identified medical journals with a 2015 impact factor of ≥15.0 on 24 May 2017, then excluded from the analysis journals that only publish review articles. Between 29 June 2017 and 26 July 2017, we collected information about each journal's open access policies from their websites and/or by email contact. We contacted the journals by email again between 6 December 2017 and 2 January 2018 to confirm our findings.

Results

Thirty-five medical journals publishing original research from 13 publishers were included in the analysis. All 35 journals offered some form of open access allowing articles to be free-to-read, either immediately on publication or after a delay of up to 12 months. Of these journals, 21 (60%) provided immediate open access with a CC BY licence under certain circumstances (e.g. to specific research funders). Of these 21, 20 only offered a CC BY licence to authors funded by non-commercial organizations and one offered this option to any funder who required it.

Conclusions

Most leading medical journals do not offer to authors reporting commercially funded research an open access licence that allows unrestricted sharing and adaptation of the published material. The journals' policies are therefore not aligned with open access declarations and guidelines. Commercial research funders lag behind academic funders in the development of mandatory open access policies, and it is time for them to work with publishers to advance the dissemination of the research they fund.

For peer review only

Strengths and limitations of this study

- This manuscript includes a cross-sectional analysis of open access policies of medical journals with a high impact factor, including society-owned journals, from multiple publishers.
- The open access policies of all journals analysed were clarified, and confirmation of our findings was received by email from 97% of the contacted journals.
- Open access policies of the journals and publishers analysed are subject to change, so the information presented here may change in the future.
- By selecting journals with a high impact factor, our analysis does not include prestigious journals from specialized therapy areas and regional or non-English language journals, which may have lower impact factors.
- Some of the journals included in our analysis (e.g. *Science*, *Nature*) could be considered as interdisciplinary journals rather than exclusively medical journals.

INTRODUCTION

Hundreds of billions of US dollars are invested in medical research by governments, charities, and philanthropic and commercial organizations each year, with the aim of extending and improving human lives.[1] Publication plays an important role in the dissemination of scientific innovation.[2 3] However, translation of medical research into clinical practice is slow; one study has suggested that it takes an average of 17 years for research evidence to reach 50% adoption in clinical practice, with the longest delays occurring after successful publication of clinical trial results.[2 3] Implementation of research published using the traditional subscription publication model is hindered by copyright restrictions that prohibit reuse of the published content and paywalls that prevent public access.

Open access publishing has the potential to improve innovation and speed up its adoption. Complete access to research literature encourages viewing of more articles than partial access,[4 5] and open access articles appear to be downloaded more often and receive more citations than subscription articles, indicating a greater

For peer review only

academic impact.[6-9] There is also evidence suggesting that open access articles have a broader societal impact based on altmetric data that measure the attention publications receive in the news and social media.[9-11] Depending on the restrictiveness of its licensing, open access can facilitate public and commercial reuse of research results, which is beneficial for collaboration, education and innovation.[9]Access to the full text of research articles also increases transparency, benefitting the public by helping both doctors and patients to find complete and current evidence to inform treatment decisions, and by preventing potentially harmful decisions being made based on the abstracts of paywalled articles.[9, 12-14] The publishing model used by a journal (i.e. open access or subscription) has no impact on the quality of articles published.[15, 16]

'Open access' is a broad term that encompasses a range of definitions, from 'free-to-read' (full text available to read on demand, without charge to the reader) to 'free-to-read and reuse' (with the additional ability to reuse text, tables and figures in different formats). The Budapest Open Access Initiative,[17] the Berlin Declaration,[18] the Bethesda Statement[19] and open access advocates[20] define 'open access' exclusively as published content that can be read free-of-charge immediately at the time of publication with unrestricted reuse rights providing that the original source is attributed. Therefore, these open access advocates and declarations recommend open access publishing under a Creative Commons Attribution (CC BY) licence, which allows sharing and adaptation of published materials for any purposes (both commercial and non-commercial), subject only to attribution of the original source.[17, 21, 22] Common alternatives to the CC BY licence include CC BY Non-Commercial (CC BY-NC), which restricts commercial reuse; CC BY No Derivatives (CC BY-ND), which restricts adaptation; and CC BY-NC-ND, which restricts both (table S1).[21, 23]

When a journal offers open access, it has wide scope in the choice of policy or policies it will apply, using one of the Creative Commons licences that allow reuse under specific terms, or offering free-to-read access without a licence.[21] The Directory of Open Access Journals (DOAJ) requires journals indexed in the directory to state on their websites clearly and precisely the terms of use and reuse that readers and authors have when they submit an article or use the published content.

For peer review only - http://bmjopen.bmj.com/site/about/guidelines.xhtml

DOAJ has a strong preference for the use of Creative Commons licenses, especially the CC BY licence.[24]

At prominent journals, open access with a Creative Commons licence is typically facilitated by an article processing charge. Following payment by the research author, institution or funder, articles are usually made available on the journal's website at the time of publication in the publisher's typeset format (Version of Record). Open access articles that do not include a Creative Commons licence at the time of publication typically involve an embargo period before the published articles are freely accessible and may allow access only to the accepted manuscript (a version that has not been edited and typeset by the journal), which is made available on the author's institutional website, PubMed Central or Europe PubMed Central without a requirement for payment. It is noteworthy that the accepted version of a manuscript as well as the Version of Record are sometimes required to bear a Creative Commons licence, often including the -NC and/or -ND clause.[25]

There has been an increasing trend towards open access publishing over the last 20 years, and almost 50% of articles were published open access in 2015.[8] However, a study analysing global health research articles published between 2010 and 2014 showed that 69% of these articles were not freely available on the journal's website and 61% of researchers do not self-archive their work even when journal policy allows them to do so free of charge.[26] Many academic and not-for-profit research funders now require the research they fund to be published open access.[9] [27-32] Prominently, the Wellcome Trust and the Bill & Melinda Gates Foundation insist on publishing with a CC BY licence to allow the broadest possible use.[27 29] Commercial research funders, which fund approximately half of all medical research,[1 33 34] have been more hesitant to require open access publishing but now commonly pay for open access when the option is available.[30] Commercial research funders are defined here as pharmaceutical companies and other medical industries that fund research for commercial purposes. The proportion of articles authored by large pharmaceutical companies that were published open access doubled between 2009 and 2016.[35] In January 2018, Shire (now part of Takeda) became the first commercial research funder to require all research manuscripts it funds to be

For peer review only - http://bmjopen.bmj.com/site/about/guidelines.xhtml

published open access.[36 37] One year later, Ipsen committed to making its published scientific research freely accessible to everyone.[38]

We set out to clarify the open access variants provided by leading medical journals for research in general, and commercially funded research in particular, and establish the availability of the CC BY licence for commercially funded research.

For peer review only

For peer review only - http://bmjopen.bmj.com/site/about/guidelines.xhtml

METHODS

Using Journal Selector (Sylogent, Newtown, PA, USA), we identified medical journals with a 2015 impact factor of at least 15.0 (accurate on 24 May 2017). To focus on journals publishing original medical research, we excluded journals that only publish review articles. We collected information on the open access variants provided by the included journals from their websites and by email contact when information was missing or unclear, making up to three attempts between 29 June 2017 and 26 July 2017.

For each journal, we recorded the following information:

- for immediate open access, whether a CC BY licence or other Creative Commons licence was provided
- for delayed open access, the length of embargo period for open access
- for both immediate and delayed open access, which version of the article would be available (published Version of Record or accepted).

For journals that provided a CC BY licence, we additionally collected information on:

- the requirements for obtaining a CC BY licence (e.g. dependence on funding source)
- article processing charges.

Between 6 December 2017 and 2 January 2018, we emailed the journals' editorial offices requesting confirmation of our findings. Once open access variants were recorded, we categorized the most open variant provided by each included journal using our own classification, as shown in table 1.

To gather general information on open access licences and charges available from a larger selection of medical journals, we carried out a search on the DOAJ website (https://doaj.org/search) on 21 February 2019.

For peer review only

Patient and public involvement

Although patients and/or the public were not directly involved in the design and conduct of this study, patients' perspectives were sought during the reporting of our findings and are included in the Supplemental information.

For peer review only

For peer review only - http://bmjopen.bmj.com/site/about/guidelines.xhtml

RESULTS

Included journals

Fifty-three journals listed in the Journal Selector database had a 2015 impact factor of at least 15.0 (figure 1). After 16 review journals and two non-medical journals were excluded, 35 journals from 13 publishers were included in this analysis. Of the 15 journals that were contacted to clarify information that was missing or unclear, 14 replied with clarification. Once all information was collected and tabulated, we received confirmation of our findings from 34 (97%) of the 35 journals.

Open access variants offered

Proportions of journals in each category of the most open variant of open access are shown in figure 2A. Immediate open access with a Creative Commons licence was provided by 21 (60%) of the 35 journals analysed. The types of Creative Commons licence available from these 21 journals under different circumstances were: CC BY from 21 journals (100%); CC BY-NC from 4 journals (19% of all journals offering CC BY); and CC BY-NC-ND from 18 journals (86% of all journals offering CC BY).

When the 35 analysed journals were categorized by impact factor, immediate open access with a CC BY or other Creative Commons licence was provided by 10 (66%) of the 15 journals with an impact factor between 15.0 and 19.9, and 3 (30%) of the 10 journals with an impact factor over 30.0 (figure 2B).

All 14 journals, from six publishers, that did not provide open access with a Creative Commons licence provided access to different versions of the article either immediately, after a 6-month embargo period or after a 12-month embargo period under different circumstances (table 2).

For peer review only

For peer review only - http://bmjopen.bmj.com/site/about/guidelines.xhtml

The cost of open access with a CC BY licence

Of the 21 journals that offered a CC BY licence, 19 (90%) disclosed article processing charges on their websites. Across these journals, charges ranged from USD $3000 to $5000; the most common article processing charge was $5000 (in 13 [62%] of journals; figure 3). Of the six journals disclosing an article processing charge of less than $5000, five had an impact factor of less than 20.0, indicating that the cost of article processing charges may depend on impact factor. Details of the fees charged by the remaining two journals (10%), *Science* and *Science Translational Medicine,* were not available from their websites because the details were only provided when the article was accepted.[39]

Relationship between funding source and the availability of open access variants

Table 3 shows the open access policies of the journals offering open access with a CC BY licence. Of the 21 journals listed, 20 journals allowed open access with a CC BY licence for research funded by specific non-commercial organizations, and only *The BMJ* offered it to any organization who required it, regardless of the nature of the funding source.

Availability of open access from a larger selection of medical journals

Of 713 medical journals indexed in the DOAJ database on 21 February 2019: 689 (96.6%) supported publishing with a Creative Commons licence and 24 (3.4%) supported publishing with the publisher's own licence; 227 (31.8%) journals charged article processing charges for open access; and 257 (36.0%) journals listed CC BY as their most restrictive licence regardless of whether there was an associated article processing charge. Of the 257 journals that allowed open access publishing with a CC BY licence, 108 (42.0%) charged an article processing charge for the opportunity and two (0.8%) did not have available information on publication charges.

DISCUSSION

Here, we present a cross-sectional analysis of open access policies of medical journals with a high impact factor, including society-owned journals, from multiple publishers. We met our objective to clarify the open access policies of all journals

For peer review only - http://bmjopen.bmj.com/site/about/guidelines.xhtml

analysed and received confirmation of our findings by email from 97% of the contacted journals. We found that all leading medical journals in this study provided some form of open access, but there was little consistency across their policies. Over half of the included journals provided a CC BY licence; however, with the exception of one journal, this option was available only to authors funded by non-commercial organizations. One journal (*The BMJ*) allowed authors to obtain a CC BY licence when the work was supported by funders who required its use. Therefore, if commercial organizations, such as pharmaceutical companies had a policy that required open access with a CC BY licence, *The BMJ* would currently be the only compliant medical journal with an impact factor greater than 15.0. At the time of our analysis, no commercial research funder required open access with a CC BY licence. However, the company at which the analysis was performed, Oxford PharmaGenesis, has since updated its publication policy to require open access with a CC BY licence for the research it funds.[40]

Limitations of this study are that we investigated journals listed in the Journal Selector database with an impact factor of at least 15.0, and that, because impact factors and the open access policies of journals and publishers are subject to change, the information may change in the future. The validity of the impact factor metric is contentious, and its use in this analysis may have led to exclusion of prestigious journals from specialized therapy areas and regional or non-English language journalsthat have impact factors under 15.0. Furthermore, some of the journals included in our analysis (e.g. *Science*, *Nature*) can be considered interdisciplinary journals rather than exclusively medical journals. Although our study covers only a small number of journals, extending such a manual analysis to a greater number of journals without loss of detail and verification of all results would take more time and increase the scope of the study. If more extensive mining of journal metadata becomes feasible, , this study could be more easily repeated for a bigger cohort of journals. To gather general information on open access licences and charges available from a larger selection of medical journals, we carried out a search on the DOAJ website on 21 February 2019. Unlike our manual analysis of medical journals with a high impact factor, the search of medical journals indexed in the DOAJ included only those that met the DOAJ criteria to be considered an open access journal. Therefore, it is not surprising that the proportion of journals that

For peer review only - http://bmjopen.bmj.com/site/about/guidelines.xhtml

provided open access with a Creative Commons licence in our manual analysis (60.0%) was lower than that found in the DOAJ search (96.6%). To our surprise, the proportion of journals that provided open access with a CC BY licence was higher for the journals in our manual analysis (60.0%) than for the journals identified in the DOAJ search (36.0%). However, the DOAJ currently only lists one licence for each journal and asks publishers to choose the most restrictive licence, so there is a possibility that the CC BY licence is available from a greater proportion of medical journals indexed in the DOAJ. In our manual analysis, all included medical journals that provided open access with a CC BY licence required payment of an article processing charge, but less than half of the medical journals in the DOAJ charge for a CC BY licence. This finding suggests that medical journals with a high impact factor charge more for publishing open access with a CC BY licence than the average medical open access journal. Information on whether the availability of the CC BY licence is dependent on the funding source could not be easily found using the DOAJ search.

To our knowledge, this is the first report showing that the availability of open access options depends on the source of funding. A previous study by Solomon and Björk analysing the source of funding for open access publishing across 74 open access journals of different disciplines showed that 50% of the open access publications in Health Sciences, Biology, and Life Sciences were funded by a grant/contract or national funding and 30% of the publications were funded by an institution.[41] However, the study did not show that the availability of open access was dependent on whether the source of funding is commercial or non-commercial.[41] In line with our results, the analysis by Solomon and Björk showed that journals with the highest impact factor tended to charge the highest article processing charges.[41] Limitations on the availability of the CC BY licence depending on the research funder are not in line with statements such as the Budapest Declaration,[17] the Berlin Declaration[18] and the Bethesda Statement,[19] which aim to provide end users with immediate access to research articles and to give them the opportunity to reuse material without restrictions. Furthermore, placing restrictions on access to medical research owing to its source of funding is not in line with the key principles of human research ethics laid out in the Declaration of Helsinki.[42]

For peer review only - http://bmjopen.bmj.com/site/about/guidelines.xhtml

Good Publication Practice 3 (GPP3) guidelines state that authors should take responsibility for the way research findings are published.[43] In line with these recommendations, commercial companies can and, we believe, should advise authors to reach a consensus on which journal to publish with, to avoid predatory journals, and to adhere to sponsor guidelines and regulations. In the authors' experience, some pharmaceutical companies already have internal guidelines recommending open access publishing, and two (Shire, now part of Takeda, and Ipsen) now requires it.[37 38]

Our research shows that one-third of the journals with a high impact factor do not offer immediate access to the published version of a manuscript upon publication, even though the open access policies of many funders with respect to embargo periods echo the recommendations set out by open access declarations worldwide.[17-19 27-29 32 44] Of note, Horizon 2020, which is supported by the European Research Council, requires its beneficiaries to make publications open access no later than 6 months after the official publication date and to make every effort to allow for maximum reuse of the materials, whether that be copying, distributing, searching, linking, crawling, mining or some other use.[45 46] Furthermore, cOAlition S, a group of national research funders with the support of the European Commission and the European Council, has committed to Plan S, the key principle of which is that scientific publications on research funded by participating national and European funders must be published open access by 2020.[44] Under the terms of Plan S, authors must retain copyright of their publication with no restrictions, and all publications must be published under an immediate open licence (preferably CC BY) that fulfils the requirements defined by the Berlin Declaration.[44 45]

Policies vary between publishers but also across journals at the same publisher, and this is also the case for journals not included in this analysis, as shown, for example, by Taylor & Francis in their table of the policies of all their journals.[47] Differences in policy have many underlying factors, including the choices of the journals' academic editorial boards and societies. A potential disincentive to publishers offering CC BY licences to commercial research funders is the revenue generated from copyright fees and reprints. Permission to reproduce copyrighted materials can cost hundreds or even thousands of dollars; for example, the permission fee requested for reuse of

For peer review only - http://bmjopen.bmj.com/site/about/guidelines.xhtml

a single table containing 40 words in the journal *American Family Physician* was $4400.[48] Reprints can cost significantly more than permissions charges; for example, reprint sales from a single clinical trial can total $1 million or more, with a large profit margin.[49]

Research by Lundh *et al.*[50] aimed to quantify reprint revenues as a proportion of journal income. Of the six journals investigated, the two European journals, *The BMJ* and *The Lancet*, owned by Elsevier, disclosed the information requested. The editors of the US journals *Archives of Internal Medicine*, *Annals of Internal Medicine*, *JAMA* and the *New England Journal of Medicine* did not provide the data. For *The BMJ*, reprint revenues constituted 3% of its overall income; *The Lancet* obtained 41% of its revenue from reprints.[50] In *The Lancet*, commercially funded publications constituted a large proportion of highly reprinted articles (63/88) compared with a sample of control articles from the same journal (23/88).[51] The generation of revenue for publishers from the selling of reprints leaves publishers open to the criticism that bias can be introduced into editorial decisions.[50] This concern could be addressed by a transition to open access publishing exclusively with a CC BY licence.

Two of the journals included in our analysis, *Science* and *Science Translational Medicine*, both published by the American Association for the Advancement of Science, do not disclose article processing charges on their websites;[39] instead, they provide this information upon their acceptance of an article. This practice does not comply with the DOAJ guidelines,[52] which state that processing fees must be stated clearly on journal websites in a place that is easy to find for potential authors prior to submitting their manuscript.

We found that the open access policies of some journals precluded commercially funded research from being published open access, even after an embargo period and without a Creative Commons licence. Further analyses could therefore be undertaken to clarify the proportion of journals with this policy and the rationale behind this position. Future research could also focus on a larger cohort of journals than the current study, or on journals from a specific therapy area, to clarify further the use of open access variants in the medical publications landscape. Future work could also involve collecting information on whether medical journals with a high

For peer review only - http://bmjopen.bmj.com/site/about/guidelines.xhtml

impact factor allow commercial funders to use preprints or registered reports, which speed up research dissemination and remove publication bias, respectively. For example, it would be interesting to see whether journals that do not provide immediate open access options to commercial funders allow research manuscripts to be posted as preprints, and therefore support immediate dissemination of the results, albeit in a manuscript that has not yet been peer reviewed.

CONCLUSIONS

The CC BY licence is recommended by open access declarations and funders of research as the optimal open access licence. Our analysis shows that although medical journals with a high impact factor provide some form of open access, they restrict commercially funded research from being published with the CC BY licence, meaning that the research output cannot be reused or built upon if it is published in journals with a high impact factor without payment of additional fees. These restrictions hamper the further development and implementation of the approximately half of all medical research that is funded by commercial research funders.[1 33 34]

Open access publishing facilitates faster and more thorough disclosure of research, removes barriers for groups conducting systematic reviews, increases both the citation counts and altmetric scores of publications, and benefits patient health by improving informed decision-making by doctors and patients.[9] Commercial research funders lag behind non-commercial funders in the implementation of open access policies, and we believe that it is time for them to close the gap. Commercial companies could, and we believe should, make clear their open access requirements, for example in a unified position statement, ideally aligned with open access declarations,[17-19] the Horizon 2020 programme and Plan S,[44-46] and the International Committee of Medical Journal Editors[53] and GPP3[43] guidelines, and then work together with publishers to realise the ultimate goal of improved access to medical research for all.

For peer review only

Acknowledgements

Robert Kiley (https://orcid.org/0000-0003-4733-2558) is Head of Open Research at the Wellcome Trust, London, UK, and contributed to the review of this manuscript. Paul Farrow (https://orcid.org/0000-0002-0569-9688) is an employee of Oxford PharmaGenesis, Oxford, UK, and contributed significantly to the review of this manuscript. Sarah Stokes (https://orcid.org/0000-0002-8761-8588) and Velissaria Vanna are employees of Oxford PharmaGenesis, Oxford, UK, and contributed to the review and editing of this manuscript. The authors also thank Alan Thomas and Elizabeth Kinder for their review of this article from the patient perspective. This work was presented as a poster at both the European Meeting of the International Society for Medical Publication Professionals (ISMPP) on 23 January 2018 and the Annual Meeting of ISMPP on 2 May 2018, and was posted to bioRxiv as a preprint on 22 January 2018 (https://www.biorxiv.org/content/early/2018/01/22/250613).

Funding statement

This research was funded by Oxford PharmaGenesis.

Competing interests

Tim Ellison, Tim Koder and Chris Winchester are employees of Oxford PharmaGenesis, Oxford, UK. At the time of the research and writing of this manuscript, Laura Schmidt and Amy Williams were employees of Oxford PharmaGenesis, Oxford, UK and are currently employed by Comradis and dna Communications, respectively. Chris Winchester is also a Director and a shareholder of Oxford PharmaGenesis Holdings Ltd.

Author contributions

Conceptualization, project administration, TE (https://orcid.org/0000-0003-0307-725X), TK (https://orcid.org/0000-0001-6152-7365), LS (https://orcid.org/0000-0001-6117-781X), AW (https://orcid.org/0000-0002-9354-6402); methodology, resources, investigation, formal analysis, TE; writing – original draft, TE and LS; visualization, TE; writing – review and editing, TE, TK, LS, AW, CW (https://orcid.org/0000-0003-3267-3990); supervision, TK, LS.

For peer review only

Data availability statement

All data relevant to the study are included in the article or uploaded as supplementary information.

For peer review only

For peer review only - http://bmjopen.bmj.com/site/about/guidelines.xhtml

References

1. Moses H, 3rd, Matheson DH, Cairns-Smith S*, et al.* The anatomy of medical research: US and international comparisons. *JAMA* 2015;313(2):174–89. doi: 10.1001/jama.2014.15939.
2. Balas EA, Boren SA. Managing clinical knowledge for health care improvement. *Yearb Med Inform* 2000(1):65–70.
3. Balas EA, Chapman WW. Road map for diffusion of innovation in health care. *Health Aff (Millwood)* 2018;37(2):198–204. doi: 10.1377/hlthaff.2017.1155.
4. Maggio LA, Moorhead LL, Willinsky JM. Qualitative study of physicians' varied uses of biomedical research in the USA. *BMJ Open* 2016;6(11):e012846. doi: 10.1136/bmjopen-2016-012846.
5. Moorhead LL, Holzmeyer C, Maggio LA*, et al.* In an age of open access to research policies: physician and public health NGO staff research use and policy awareness. *PLoS One* 2015;10(7):e0129708. doi: 10.1371/journal.pone.0129708.
6. Davis PM, Lewenstein BV, Simon DH*, et al.* Open access publishing, article downloads, and citations: randomised controlled trial. *BMJ* 2008;337:a568. doi: 10.1136/bmj.a568.
7. Ottaviani J. The post-embargo open access citation advantage: it exists (probably), its modest (usually), and the rich get richer (of course). *PLoS One* 2016;11(8):e0159614. doi: 10.1371/journal.pone.0159614.
8. Piwowar H, Priem J, Lariviere V*, et al.* The state of OA: a large-scale analysis of the prevalence and impact of Open Access articles. *PeerJ* 2018;6:e4375. doi: 10.7717/peerj.4375.
9. Tennant JP, Waldner F, Jacques DC*, et al.* The academic, economic and societal impacts of open access: an evidence-based review. *F1000Res* 2016;5:632. doi: 10.12688/f1000research.8460.3.
10. Wang X, Liu C, Mao W*, et al.* The open access advantage considering citation, article usage and social media attention. *Scientometrics* 2015;103(2):555–64. doi: 10.1007/s11192-015-1547-0.

For peer review only

11. Allen HG, Stanton TR, Di Pietro F*, et al.* Social media release increases dissemination of original articles in the clinical pain sciences. *PLoS One* 2013;8(7):e68914. doi: 10.1371/journal.pone.0068914.

12. Hopewell S, Clarke M, Moher D*, et al.* CONSORT for reporting randomised trials in journal and conference abstracts. *Lancet* 2008;371(9609):281–3. doi: 10.1016/S0140-6736(07)61835-2.

13. Barbour V, Chinnock P, Cohen B*, et al.* The impact of open access upon public health. *Bull World Health Organ* 2006;84(5):337–424.

14. Leung PTM, Macdonald EM, Stanbrook MB*, et al.* A 1980 letter on the risk of opioid addiction. *N Engl J Med* 2017;376(22):2194–95. doi: 10.1056/NEJMc1700150.

15. Pastorino R, Milovanovic S, Stojanovic J*, et al.* Quality assessment of studies published in open access and subscription journals: results of a systematic evaluation. *PLoS One* 2016;11(5):e0154217. doi: 10.1371/journal.pone.0154217.

16. Tahim A, Bansal H, Goodson AM*, et al.* Open access publishing: a study of current practice in oral and maxillofacial surgery research. *J Maxillofac Oral Surg* 2016;15(4):517-20. doi: 10.1007/s12663-016-0898-2.

17. Budapest Open Access Initiative. Ten years on from the Budapest Open Access Initiative: setting the default to open 2012. Available from: http://www.budapestopenaccessinitiative.org/boai-10-recommendations (Accessed 2 January 2018).

18. Open access Max-Planck-Gesellschaft. Berlin Declaration on Open Access to Knowledge in the Sciences and Humanities 2003. Available from: https://openaccess.mpg.de/Berlin-Declaration (Accessed 2 January 2018).

19. Brown PO, Cabell D, Chakravarti A*, et al.* Bethesda Statement on Open Access Publishing Chevy Chase, Maryland, US 2003. Available from: http://legacy.earlham.edu/~peters/fos/bethesda.htm (Accessed 3 January 2018).

20. Carroll MW. Why full open access matters. *PLoS Biol* 2011;9(11):e1001210. doi: 10.1371/journal.pbio.1001210.

21. Creative Commons. About The Licenses: Creative Commons; 2017. Available from: https://creativecommons.org/licenses/ (Accessed 29 October 2018).

22. Williams L. Best practices in licensing and attribution: What you need to know: OASPA; 2016. Available from: https://oaspa.org/best-practices-licensing-attribution-need-to-know/ (Accessed 3 January 2018).

For peer review only

23. Stodden V, Leisch F, Peng RD. Implementing Reproducible Research: Taylor & Francis 2014.
24. Directory of Open Access Journals. Information for publishers. Available from: https://doaj.org/publishers#licensing (Accessed 21 February 2019).
25. Elsevier. Open access licences. Available from: https://www.elsevier.com/about/policies/open-access-licenses (Accessed 21 February 2019).
26. Smith E, Haustein S, Mongeon P*, et al.* Knowledge sharing in global health research - the impact, uptake and cost of open access to scholarly literature. *Health Res Policy Syst* 2017;15(1):73. doi: 10.1186/s12961-017-0235-3.
27. Open access policy: Wellcome Trust. Available from: https://wellcome.ac.uk/funding/managing-grant/open-access-policy (Accessed 3 January 2018).
28. Charity Open Access Fund. COAF guidelines: Charity Open Access Fund; 2017. Available from: https://wellcome.ac.uk/funding/managing-grant/charity-open-access-fund (Accessed 2 January 2018).
29. Bill & Melinda Gates Foundation. How We Work: Bill & Melinda Gates Foundation Open Access Policy 2015. Available from: https://www.gatesfoundation.org/How-We-Work/General-Information/Open-Access-Policy (Accessed 3 January 2018).
30. Collins E. Publishing priorities of biomedical research funders. *BMJ Open* 2013;3(10):e004171. doi: 10.1136/bmjopen-2013-004171.
31. Marchington J, Panayi A, Baronikova S*, et al.* Open access licences: what drives publisher options? Suppl 1 (26)2017. Available from: http://www.caudex.com/downloads/OA_survey_EU_ISMPP_2017_poster_15.pdf (Accessed 3 January 2018).
32. Medical Research Council UK. RCUK Policy on Open Access and Supporting Guidance 2017. Available from: http://www.rcuk.ac.uk/documents/documents/rcukopenaccesspolicy-pdf/ (Accessed 2 January 2018).
33. Dorsey ER, de Roulet J, Thompson JP*, et al.* Funding of US biomedical research, 2003–2008. *JAMA* 2010;303(2):137–43. doi: 10.1001/jama.2009.1987.
34. Hakoum MB, Jouni N, Abou-Jaoude EA*, et al.* Characteristics of funding of clinical trials: cross-sectional survey and proposed guidance. *BMJ Open* 2017;7(10):e015997. doi: 10.1136/bmjopen-2017-015997.

For peer review only - http://bmjopen.bmj.com/site/about/guidelines.xhtml

35. Yegros-Yegros A, van Leeuwen T. Production and uptake of Open Access publications involving the private sector: the case of big pharma (preprint). *SocArXiv* 2019 doi: https://doi.org/10.31235/osf.io/zt6kc
36. The MAP Newsletter. Shire announces new open access policy: ISMPP; 2018 updated 20 January 2018. Available from: http://ismpp-newsletter.com/2018/01/30/shire-announces-new-open-access-policy/ (Accessed 20 July 2018).
37. Shire. Shire continues to uphold high standards of ethics and transparency with adoption of open access policy for publication of Shire-supported research 2018. Available from: https://www.shire.com/en/newsroom/2018/january/xajhds (Accessed 16 July 2018).
38. Ipsen commits to making all its published scientific research freely accessible to everyone: Ipsen. Available from: https://www.ipsen.com/ipsen-commits-to-making-all-its-published-scientific-research-freely-accessible-to-everyone/ (Accessed 25 February 2019).
39. Science Journals: editorial policies: *Science*. Available from: http://www.sciencemag.org/authors/science-journals-editorial-policies (Accessed 14 September) 2018.
40. Oxford PharmaGenesis launches open access publication policy: Oxford PharmaGenesis. Available from: https://www.pharmagenesis.com/oxford-pharmagenesis-launches-open-access-publication-policy/#.XHP-LIj7SUk (Accessed 25 February 2019).
41. Solomon DJ, Björk B. Publication fees in open access publishing: sources of funding and factors influencing choice of journal. *Journal of the American Society for Information Science & Technology* 2012;63(1):98–107.
42. WMA Declaration of Helsinki – Ethical principles for medical research involving human subjects: World Medical Association. Available from: https://www.wma.net/policies-post/wma-declaration-of-helsinki-ethical-principles-for-medical-research-involving-human-subjects/ (Accessed 19 January) 2018.
43. Battisti WP, Wager E, Baltzer L, *et al.* Good Publication Practice for Communicating Company-Sponsored Medical Research: GPP3. *Ann Intern Med* 2015;163(6):461–4. doi: 10.7326/M15-0288.
44. Science Europe. cOAlition S 2018. Available from: https://www.scienceeurope.org/coalition-s/ (Accessed 29 October 2018).

For peer review only - http://bmjopen.bmj.com/site/about/guidelines.xhtml

45. Guidelines to the rules on open access to scientific publications and open access to research data in Horizon 2020 (Version 3.2): EUROPEAN COMMISSION Directorate-General for Research & Innovation; 2017. 11. Available from: http://ec.europa.eu/research/participants/data/ref/h2020/grants_manual/hi/oa_pilot/h2020-hi-oa-pilot-guide_en.pdf (Accessed 17 January 2018).

46. Guidelines on the Implementation of Open Access to Scientific Publications and Research Data in projects supported by the European Research Council under Horizon 2020: European Research Council 2017. Available from: https://erc.europa.eu/sites/default/files/document/file/ERC%20Open%20Access%20guidelines-Version%201.1._10.04.2017.pdf (Accessed 3 January 2018).

47. Author Services Supporting Taylor & Francis authors. Open access options: Taylor & Francis. Available from: https://authorservices.taylorandfrancis.com/journal-list/ (Accessed 20 July 2018).

48. Siwek J. Permission fees for reproducing tables in journal articles are exorbitant. *BMJ* 2015;351:h5128. doi: 10.1136/bmj.h5128.

49. Smith R. Medical journals and pharmaceutical companies: uneasy bedfellows. *BMJ* 2003;326(7400):1202–5. doi: 10.1136/bmj.326.7400.1202.

50. Lundh A, Barbateskovic M, Hrobjartsson A, *et al.* Conflicts of interest at medical journals: the influence of industry-supported randomised trials on journal impact factors and revenue – cohort study. *PLoS Med* 2010;7(10):e1000354. doi: 10.1371/journal.pmed.1000354.

51. Handel AE, Patel SV, Pakpoor J, *et al.* High reprint orders in medical journals and pharmaceutical industry funding: case-control study. *BMJ* 2012;344:e4212. doi: 10.1136/bmj.e4212.

52. Principles of transparency and best practice in scholarly publishing: DOAJ. Available from: https://blog.doaj.org/2018/01/15/principles-of-transparency-and-best-practice-in-scholarly-publishing-version-3/ (Accessed 18 January) 2018.

53. International Committee of Medical Journal Editors. Recommendations for the conduct, reporting, editing, and publication of scholarly work in medical journals: International Committee of Medical Journal Editors; 2017. Available from: http://www.icmje.org/icmje-recommendations.pdf (Accessed 2 January 2017).

For peer review only - http://bmjopen.bmj.com/site/about/guidelines.xhtml

Tables

Table 1 Categorization of journals based on the most open variant of open access offered.

Category	Version of article available	Embargo period*	CC BY licence offered by the journal?
**1**	Published	None	Yes
**2**	Published	None	No
**3**	Published/accepted	≤ 12 months	No

*None=immediate open access; > 0 months=delayed open access.

CC BY, Creative Commons Attribution.

For peer review only

Table 2 Access policies of journals with high impact factors that do not provide open access with Creative Commons licences.

Publisher	Organisation status	Journals included (n=14)	Open access variants available*	
			Embargo period†	Version of article available
**American Association for Cancer Research journals**	Nonprofit society	*Cancer Discov*	None	VoR‡
			6–12 months	Accepted
**American College of Physicians**	Nonprofit society	*Ann Intern Med*	6 months	Accepted
**American Medical Association**	Nonprofit society	*JAMA*	None	VoR§
			6 months	VoR
**Massachusetts Medical Society**	Nonprofit society	*N Engl J Med*	6 months	VoR
**Nature Publishing Group**	Commercial	*Nature*; *Nat Biotechnol*; *Nat Cell Biol*; *Nat Genet*; *Nat Immunol*; *Nat Mater*; *Nat Med*; *Nat Methods*; *Nat Neurosci*	6 months	Accepted

For peer review only - http://bmjopen.bmj.com/site/about/guidelines.xhtml

**Wiley-Blackwell**	Commercial	*World Psychiatry*	12 months	Accepted

*Available under the terms specified on the journal website.

†None=immediate open access; > 0 months=delayed open access.

‡Upon payment of USD $3500 AuthorChoice fee.

§Available to read on JAMA Network Reader.

VoR, version of record.

For peer review only

For peer review only - http://bmjopen.bmj.com/site/about/guidelines.xhtml

Table 3. Open access policies of journals with high impact factors that offer immediate open access with the CC BY licence (n=21) Creative Commons licences are shaded.

Publisher	Organisation status	Journals included (n=21)	Open access variants available*			Funding requirements for obtaining open access with a CC BY licence
			**Embargo period†**	**Creative Commons licence**	**Version of article available**	
**American Association for the Advancement of Science**	Nonprofit society	*Science*; *Sci Transl Med*	None	CC BY	VoR	The American Association for the Advancement of Science "will allow authors funded by the Bill & Melinda Gates Foundation to publish their research with a CC BY licence"‡
			None	None	Accepted	
			6 months	None	Accepted	
			12 months	None	VoR	
**American Society of Clinical Oncology**	Nonprofit society	*J Clin Oncol*	None	CC BY<br>CC BY-NC-ND	VoR	Creative Commons licences available only if funders are "academic institutions, not-for-profit organizations, philanthropic foundations or government agencies"
			6 months	None	VoR	
			12 months	None	VoR	
**BMJ Publishing Group**	Non-profit society	*BMJ*	None	CC BY<br>CC BY-NC	VoR	CC BY licence available for authors "where the funder requires it"
**Cell Press**	Commercial	*Cancer Cell*; *Cell*;	None	CC BY<br>CC BY-NC-ND	VoR	Creative Commons licences "available only to authors covered by a funding body agreement"

		Cell Metab; *Cell Stem Cell*; *Immunity*	12 months	None	Accepted	(these non-commercial funding bodies are listed on the journal websites)
**Elsevier**	Commercial	*Eur Urol*; *Gastroenterology*; *J Am Coll Cardiol;* *Lancet*; *Lancet Diabetes Endocrinol*; *Lancet Infect Dis*; *Lancet Oncol*; *Lancet Neurol*; *Lancet Respir Med*	None	CC BY<br>CC BY-NC-ND	VoR<br>Accepted§/VoR	Creative Commons licences are available to authors funded by specific funding bodies (these non-commercial funding bodies are listed on the journal websites)<br><br>Elsevier has established agreements and developed policies to allow authors who publish in Elsevier journals to comply with manuscript archiving requirements of various funding bodies (these non-commercial funding bodies are listed on the journal websites)
			6 months	None	VoR	

For peer review

**European Society of Cardiology**	Nonprofit society	*Eur Heart J*	None	CC BY<br>CC BY-NC<br>CC BY-NC-ND	VoR	"RCUK/Wellcome Trust-funded authors…can use the CC BY licence for their articles"
			None	None	Accepted	
			12 months	None	Accepted	
**Lippincott Williams & Wilkins**	Commercial	*Circulation*	None	CC BY<br>CC BY-NC<br>CC BY-NC-ND	VoR	"Note that authors funded by RCUK or the Wellcome Trust may choose the CC BY licence if they agree to pay the article processing charge and commercial reuse of the article is not a factor"
			6–12 months	None	Accepted	
**Wiley-Blackwell**	Commercial	*CA Cancer J Clin*	None	CC BY<br>CC BY-NC<br>CC BY-NC-ND	VoR	"All RCUK and Wellcome Trust-funded authors will be directed to the CC BY licence"
			12–24 months	None	Accepted	

*Available under the terms specified on the journal website.

†None = immediate open access; > 0 months = delayed open access.

‡The American Association for the Advancement of Science's pilot open access partnership with the Gates Foundation concluded on 30 June 2018.[39]

§Accepted manuscripts can be self-archived and are required to attach a CC BY-NC-ND licence.[25]

CC BY, Creative Commons Attribution; NC, Non-Commercial; ND, No Derivatives; RCUK, Research Councils UK; VoR, version of record.

For peer review only

Figure legends

Figure 1 Flow chart of journals included in this study.

Figure 2 Medical journals categorized by impact factor and their most open variant of open access available (n=35). **(A)** Impact factor, ≥15.0; **(B)** Impact factors, 15.0–19.9, 20.0–29.9 and ≥30.0.

CC BY, Creative Commons Attribution licence.

Figure 3 Article processing charges of journals that offer immediate open access with a CC BY licence (n=21).

*Details on processing fees are provided at acceptance.[39]

For peer review only

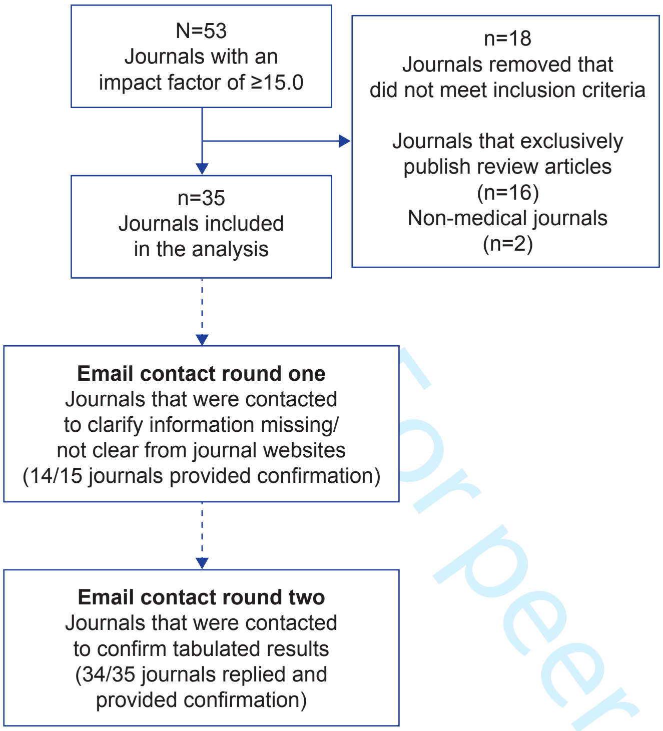

For peer review only

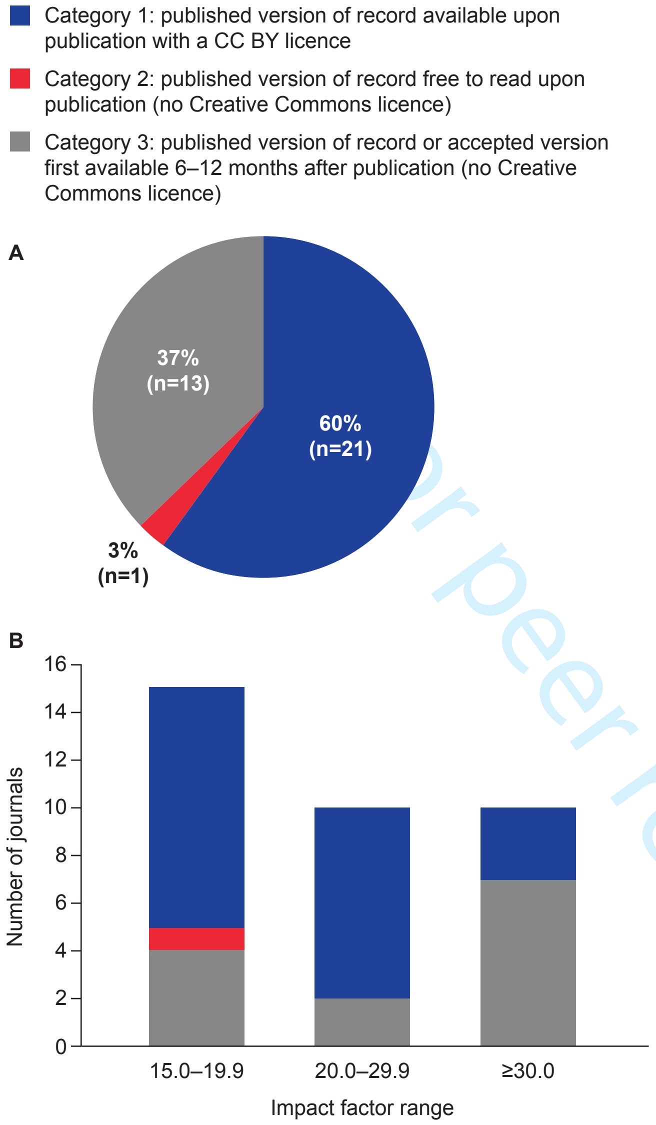

For peer review only

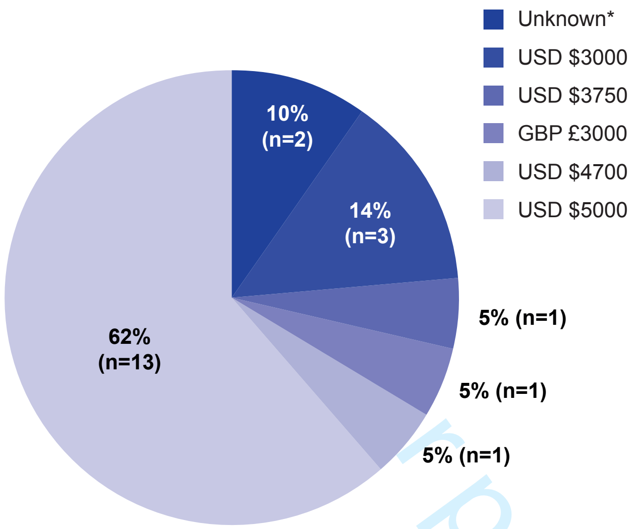

For peer review only

Supplemental information

Table S1 Definitions of CC licences commonly used by medical journals[17]

Type	Definition
**CC BY**	Free to distribute and adapt the original work, even commercially, if the original creation and authors are credited
**CC BY-NC**	Free to adapt the original work non-commercially and, although derivative works must also acknowledge the authors and be non-commercial, they do not have to be licensed on the same terms
**CC BY-NC-ND**	Free to download the original work and share it if the authors are credited, but the work cannot be adapted or used commercially

CC, Creative Commons; CC BY, Creative Commons Attribution; NC, Non-Commercial; ND, No Derivatives.

Patients' perspectives

After reviewing a draft of our manuscript, Alan Thomas, a patient advocate for rare diseases, shared his experiences. He said, "I regularly encounter paywalls while trying to find high quality and important information on a rare disease for myself and to share with other patients." Because patients can build up substantial understanding during a lifetime living with their rare disease, they are often able to read and understand articles in peer-reviewed journals. However, "when we hit a paywall, we typically give up and look for information elsewhere," he said. Elizabeth Kinder, a patient with multiple sclerosis, highlighted the importance of patients knowing about the latest research to make informed choices about treatments. She said "It is absolutely essential that validated scientific findings are as easily and freely available as all the dangerous false-hope-fake-news. A friend of mine spent his life savings on a pseudo-scientific procedure and after a brief respite, was considerably more disabled than before. And broke. Which increased the negative impact on his health. If publishers refuse to share information they are as immoral as those snake oil salesman and as dangerous to public health. Patients like me have to become our own experts. Faced with a steadily crippling disease like multiple sclerosis, it is not just in our own interests but in the interests of society as a whole that we are able to work, to contribute, to play our part, and for our families to do so, to not become our carers."

For peer review only

Elizabeth Kinder is mindful of the risks she took when taking part in a clinical trial, saying “I think ‘Sudden death’ was no. 3 in the long list of side effects on which I signed off).” She believes that “by withholding free access to research findings, journals are scorning the funders, the researchers, and people like me (patients) who risk their lives participating in a clinical trial”. Alan Thomas noted that many patients do not realize that scientific content is put behind paywalls by the journals in which it is published, and that they could misconstrue such information as being hidden by pharmaceutical companies or others. He wanted to encourage pharmaceutical companies to be more open and collaborative by making their research open access. He said, “open access to medical documents enables communication *with* patients as well as *to* patients, and is vital to patients’ wellbeing and peace of mind”.

For peer review only
